# A rare case of brain abscess caused by *Actinomyces meyeri*

**DOI:** 10.1186/s12879-020-05100-9

**Published:** 2020-05-27

**Authors:** Ranjit Sah, Gaurav Nepal, Sanjit Sah, Sonam Singla, Priti Upadhyay, Ali A. Rabaan, Kuldeep Dhama, Alfonso J. Rodriguez-Morales, Rabindra Ghimire

**Affiliations:** 1 Tribhuvan University Institute of Medicine, Kathmandu, Nepal; 2 Medanta The Medicity, Gurgaon, Haryana India; 3 Molecular Diagnostic Laboratory, Johns Hopkins Aramco Healthcare, Dhahran, Saudi Arabia; 4 Division of Pathology, ICAR-Indian Veterinary Research Institute, Izatnagar, Bareilly, Uttar Pradesh 243 122 India; 5 Public Health and Infection Research Group, Faculty of Health Sciences, Universidad Tecnologica de Pereira, Pereira, Risaralda, Colombia; 6 Grupo de Investigación Biomedicina, Faculty of Medicine, Fundación Universitaria Autónoma de las Américas, Sede Pereira, Pereira, Risaralda, Colombia; 7 Division of Infectious Disease, East Carolina University Brody School of Medicine, Greenville, NC USA

**Keywords:** *Actinomyces meyeri*, Brain abscess, Dental health, Nepal

## Abstract

**Background:**

Brain abscesses are the rare and most severe form of actinomycosis, which usually manifests as abscesses of the occipital or parietal lobe due to direct expansion from an adjacent area, the oral cavity. In the medical literature, there are only a few reported cases of brain abscess caused by Actinomyces meyeri. In this report, we present a 35-year-old male patient who experienced an insidious headache and left-sided weakness and was diagnosed with an Actinomyces meyeri brain abscess.

**Case presentation:**

A 35-year-old Nepalese man came to our institute with the primary complaint of insidious onset of headache and left-sided weakness. His physical examination was remarkable for the left-sided weakness with power 2/5 on both upper and lower limbs, hypertonia, hyperreflexia and positive Babinski sign, with intact sensory function. Cardiac examination revealed systolic murmur with regular S1 and S2, and lung examination was normal. The patient had poor dental hygiene. Biochemistry and haematology panel were normal. Urinalysis, chest X-ray and electrocardiogram revealed no abnormality. A transthoracic echocardiogram revealed mitral regurgitation. However, there was no evidence of valvular vegetation. A magnetic resonance imaging (MRI) of the brain was performed, which showed a bi-lobed rim enhancing lesion with a conglomeration of two adjoining round lesions in the right parietal parasagittal region. Perilesional oedema resulting in mass effect over the right lateral ventricle and mid-right uncal herniation with midline shift was noted. Craniotomy was performed, and the lesion was excised. Gram staining of the extracted sample revealed gram variable filamentous rods. Creamy white, moist, confluent colonies were observed after performing anaerobic culture in chocolate agar. On the gram staining, they showed gram-positive filamentous rods. *Actinomyces meyeri* was identified based on matrix-assisted laser desorption/ionization time-of-flight (MALDI-TOF) technology*.* Based on the susceptibilities, he was successfully treated with ampicillin-sulbactam.

**Conclusions:**

In conclusion, *Actinomyces* should be considered in the differential diagnosis of brain abscess in patients with poor dental hygiene, and early diagnosis and appropriate treatment can lead to better results.

## Background

Brain abscess is characterized by an accumulation of pus in the brain parenchyma. It begins as a localized area of cerebritis and develops into an encapsulated collection of pustular material, often presenting as a mass-like lesion. Brain abscesses usually result from the spread of infection from adjacent otitis media, mastoiditis and sinusitis, or hematogenous spread from infective endocarditis, pneumonia, empyema, skin infections, liver abscess, and pelvic infections [1]. Currently, brain abscesses are rare in high-income countries, partly due to the reduction of abscesses complicating otogenic infections [2]. It is increasingly recognized in certain risk groups, including those with congenital heart disease, diabetes, alcoholics, those who have had neurosurgery or head trauma and the use of corticosteroids and other immunosuppressive conditions [2–4]. Mortality from brain abscesses continues to decline with the introduction of CT, minimally invasive stereotactic-guided aspiration, and newer antimicrobials with good central nervous system (CNS) penetration [1].

The spectrum of pathogens responsible for brain abscess varies depending on the mechanism of infection, the host’s immune status and local epidemiology. *Streptococcus* species are the most commonly identified organisms in brain abscesses, followed by anaerobes, especially when it is due to spread from contiguous site. Anaerobic infections are mainly caused by the species *Fusobacterium, Peptostreptococcus, Bacteroides, Peptococcus* and *Veillonella*. *Staphylococcus aureus* and *Staphylococcus epidermidis* are often isolated from postoperative brain abscesses. However, up to 50% or more of brain abscesses are polymicrobial [2, 4, 5]. In immunocompromised populations, Nocardia species are responsible for most cases [4, 5].

In the medical literature, there are only a few reported cases of brain abscess caused by *Actinomyces meyeri*. In this report, we present a 35-year-old male patient who experienced an insidious onset of headache and left-sided weakness and was diagnosed with *Actinomyces meyeri* brain abscess. He was successfully treated with ampicillin-sulbactam.

## Case presentation

A 35-year-old Nepalese man came to our institute with a primary complaint of headache for 3 weeks and weakness on the left side of the body for 2 weeks. The severity of the headache increased gradually and became worse a few days before the presentation. Headache was not relieved by over the counter medication. It was associated with low-grade intermittent fever, nausea, and vomiting, and was exacerbated by changes in posture. There was no photophobia, neck stiffness and convulsions. Left-sided weakness was progressive, which affected his hands and legs equally, making him bedridden. There was no history of ear / nasal discharge, cough, sinus pain, surgery or trauma. At the time of presentation, the temperature was 37.7 °C, the heart rate was 88 beats per minute, the breathing rate was 15 times per minute, the arterial haemoglobin oxygen saturation was 95%, and the blood pressure was 123/72 mmHg. During the examination, he was alert, oriented to time, place and person. There was no pallor, icterus, lymphadenopathy, cyanosis and clubbing. His muscle bulk was normal, and he had a left-sided weakness with power 2/5 on both upper and lower limbs, showing hypertonia and hyperreflexia. The left limb showed a positive Babinski sign. Sensation and co-ordination were intact, and neck rigidity was absent. Thorough cranial nerves and eye examination were healthy, and there was no noticeable change in fundoscopy. The cardiac analysis revealed systolic murmur with normal S_1_ and S_2,_ and lung examination was normal. The patient had poor dental hygiene.

**Fig. 1:**
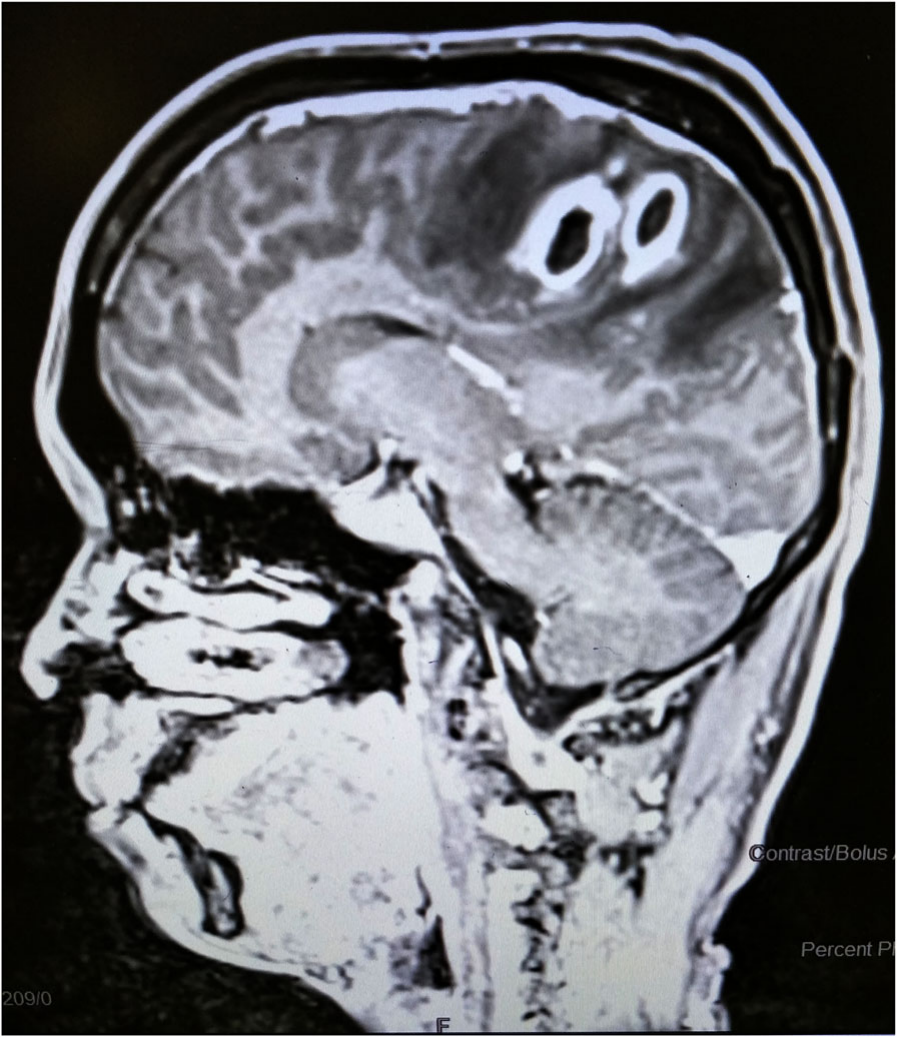
MRI of the brain showing a bi-lobed rim enhancing lesion due to conglomeration of two adjoining rounded lesion in the right parietal parasagittal region

Biochemistry and haematology panel showed normal renal and liver function, absence of leukocytosis with normal differential cell count, a normal haemoglobin level of 12.2 mg/dL, and a normal platelet count of 133,000 cells/μL. Urinalysis, chest X-ray and electrocardiogram revealed no abnormality. A transthoracic echocardiogram revealed mitral regurgitation and no evidence of valvular vegetation. A magnetic resonance imaging (MRI) of the brain was performed, which showed a bi-lobed rim enhancing lesion with a conglomeration of two adjoining round lesions in the right parietal parasagittal region (Fig. 1). Perilesional oedema resulting in mass effect over the right lateral ventricle and mid-right uncal herniation with midline shift was noted. FLAIR hyperintensity with mild leptomeningeal enhancement along the right-sided cortical sulci due to associated leptomeningitis was also reported.

**Fig. 2:**
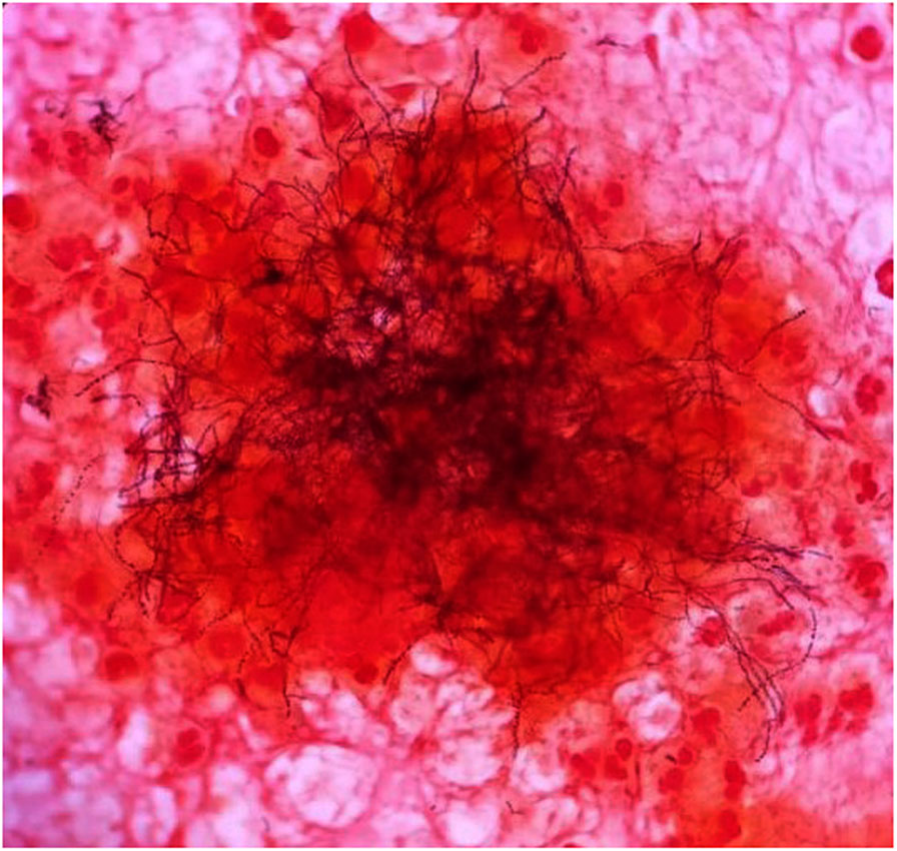
Gram stain of the excised sample revealed gram variable filamentous rods

**Fig. 3:**
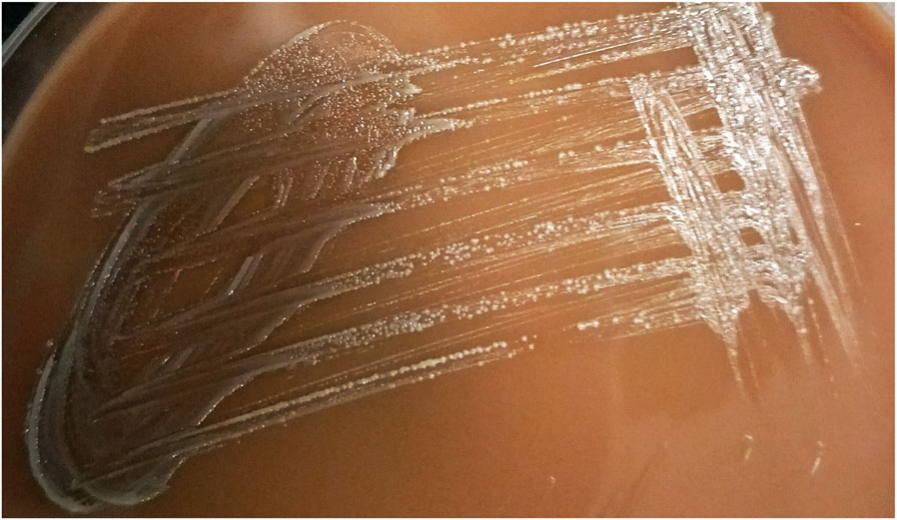
Growth on chocolate agar under anaerobic incubation showing creamy white, moist, confluent growth of the *Actinomyces meyeri*

**Fig. 4:**
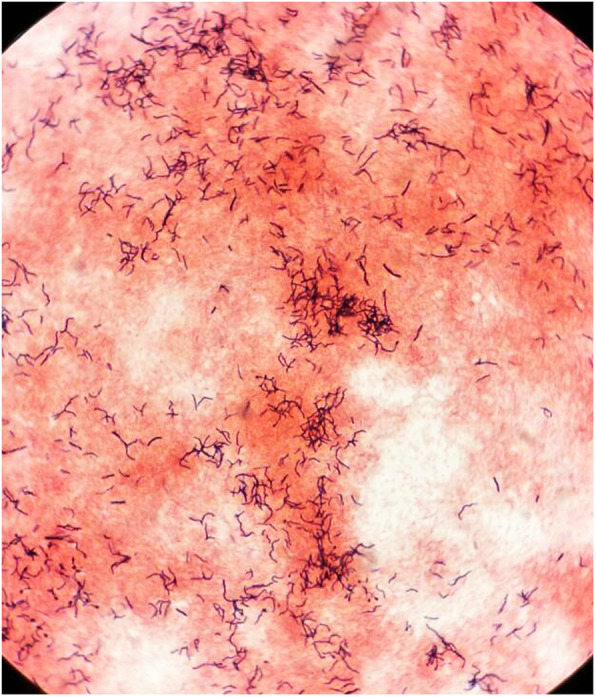
Gram stain of the isolates grown in culture showing gram-positive filamentous bacteria

Because of the treacherous mass effect of the abscess, the patient underwent craniotomy and lesion was excised, and he was empirically treated with intravenous meropenem and vancomycin. Gram stain of the extracted sample revealed gram variable filamentous rods (Fig. 2). After the gram stain results, modified acid-fast bacilli stain (modified Kinyoun staining) was performed, which was negative. There was no growth on aerobic culture, but creamy white, moist, confluent colonies were observed on chocolate agar in the anaerobic medium (Fig. 3), which on gram stain showed gram-positive filamentous rods (Fig. 4). VITEK MS, which uses MALDI-TOF technology, identified the isolated colonies as *Actinomyces meyeri.* The identification of *A. meyeri* by MALDI-TOF (VITEK® MS) had a high confidence level with 99.9% confidence value (MALDI score of more than 2), suggesting accurate identification*.* Based on the susceptibilities, antibiotics were de-escalated to ampicillin–sulbactam combination as ampicillin was not available. Headache and weakness gradually improved, and he was able to walk without assistance at 6 weeks, at which time he was discharged on oral antibiotics for 12 months.

## Discussion and conclusions

*Actinomyces meyeri* is one of many members of genus *Actinomyces*, which is normally commensal of the oropharynx, distal oesophagus and genitourinary tract, and is prone to invasive and disseminated diseases. The genus Actinomyces contains strictly anaerobic, non-acid-resistant, gram-positive bacilli / coccobacilli. An indolent course characterizes their infectious behaviour, the formation of abscesses with a woody consistency, the presence of sulfur granules and the development of a draining sinus or tract [6]. *Actinomyces meyeri* was first isolated in 1911 by Kurt F. Meyer from a sample of empyema. Till date, only about 30 cases have been recorded in the medical literature. *Actinomyces meyeri* brain abscess is even rarer; only 8 cases have been reported in the medical literature [7].

Depending on the location affected, actinomycosis is categorized as an oro-cervicofacial, thoracic, or abdominopelvic disease. Less commonly, actinomycosis can affect other parts of the body, including the musculoskeletal system, pericardium, and central nervous system [6]. Poor oral hygiene, dental procedures, aspiration of oral contents, abdominal surgery, and acute abdominal processes are identified as risk factors for actinomycosis [6, 7]. Actinomyces is particularly known for its ability to cross tissue planes [6]. Cerebral disease, i.e. brain abscesses, is a sporadic and most severe form of actinomycosis and manifests itself as abscesses of the occipital or parietal brain due to direct expansion from a contiguous site (oral cavity). However, little is known about the virulence factors that allow Actinomyces to penetrate tissues in this way [7, 8].

In our case, brain abscess was likely due to spread of *Actinomyces meyeri* from oral cavity to brain parenchyma. The oral cavity harbors more than 500 bacterial species. Just 1 mg of dental plaque may contain more than 10^11^ microorganisms. Most oral microorganisms are harmless, but if the patient’s overall health condition is weakened, even low-virulence bacteria may become harmful [9]. Odontogenic infections can cause brain abscesses in a variety of ways: 1) direct extension; 2) hematogenous spread; 3) lymphatic spread [9]. It is assumed that *actinomyces* can cause brain abscesses by direct extension [6]. A suppurative cellulitis in oral cavity can spread along the fascia1 planes to the base of the skull, the paranasal sinuses, and the orbit. Eventually, the cranial wall is penetrated by resorption of bone or the microorganisms pass through the many foramina present, causing a brain abscess [9].

The histopathological diagnosis of actinomycosis is difficult because tissue samples usually contain few sulfur granules. Also, sulfur granules may be present in nocardiosis, coccidioidomycosis, and aspergillosis [10]. Gram stain of the excised sample reveals gram variable filamentous rods. Therefore, it should be distinguished from Nocardia and Streptomyces. On an anaerobic culture, *Actinomyces meyeri* forms creamy-white, moist, confluent colonies on chocolate agar. However, cultures are negative in more than 50% of cases [10]. If culture is negative, the infection should be confirmed by other non-culture technique. In our study, we determined *Actinomyces meyeri* using MALDI-TOF [11]. If actinomyces are suspected, we recommend using MALDI-TOF instead of traditional microbiological methods. A new molecular genetic technology (massive parallel sequencing technology) using the universal amplification of bacterial 16S rRNA genes has shown that only a small percentage of bacteria present in bacterial brain abscesses can be identified by culture [12]. Traditional methods cannot detect smaller subgroups of multi-species communities present in bacterial brain abscesses. Besides, species related to the maintenance and expansion of abscesses may be missed during pus aspiration because they are mainly present near the abscess wall and not in the pus obtained by aspiration [12]. However, massive parallel sequencing technologies can characterize complex microbial communities. Kommedal et al. has found that massive parallel sequencing technology can improve the detection rate of Actinomyces species, including *A. meyeri*, compared to culture [10].

Conventional therapy for actinomycetal infections includes intravenous penicillin for 2–6 weeks, then oral penicillin or amoxicillin for an extended period of 6 to 12 months. In cases of penicillin allergy, erythromycin, tetracycline, doxycycline, minocycline and clindamycin are an alternative. However, the central nervous system permeability of all the antibiotics mentioned above is variable [7, 10]. Surgical treatment is essential for the treatment of actinomyces brain abscesses. It helps identify pathogens and their susceptibility, reduces bacterial load and surrounding stress, prevents hernias, increases oxygen tension in diseased tissue, and promotes penetration of antibiotics [7]. A previous case report of *Actinomyces israelii* brain abscess has mentioned that the aspiration of brain abscess using burr hole technique facilitate the penetration of antibiotics into the abscess cavity and may shorten the course of antibiotic therapy [8]. In our case, the surgical team opted to perform a craniotomy and excision due to right uncal herniation and midline shift and to seek a microbiological diagnosis to administer targeted antimicrobial therapy. We also chose to treat for a longer duration due to issues with follow up. However, a short course of antibiotic therapy may have been enough based on previous experience [8]. Actinomycosis may represent in many countries a challenge for specific diagnosis [12], even more in cases like this, where is presenting as a brain abscess secondary to poor dental hygiene.

In conclusion, *Actinomyces* should be considered in the differential diagnosis of brain abscess in patients with poor dental hygiene, and early diagnosis and appropriate treatment can lead to better results.

## Data Availability

Data generated or analyzed during this study are included in this published article and remaining are available from the corresponding author on reasonable request.

## Abbreviations

*A. meyeri*: Actinomyces meyeri
MALDI-TOF: Matrix-assisted laser desorption/ionization time-of-flight
MRI: Magnetic resonance imaging
CNS: Central nervous system
